# New evidence on technological acceptance model in preschool education: Linking project-based learning (PBL), mental health, and semi-immersive virtual reality with learning performance

**DOI:** 10.3389/fpubh.2022.964320

**Published:** 2022-09-13

**Authors:** Juanjuan Zang, Youngsoon Kim, Jihe Dong

**Affiliations:** ^1^Department of Pre-School Education, School of Education, Shandong Women's University, Jinan, China; ^2^Department of Social Education, College of Education, Inha University, Incheon, South Korea

**Keywords:** project-based learning, learning performance, mental health, semi-immersive virtual reality, technological acceptance

## Abstract

Despite significant research on student learning performance, the literature on preschool education is limited. The learning performance of different institutes in China has not been satisfactory, and students are not given enough technological resources to help them improve their learning performance. Although Chinese preschool students are active learners, their learning performance is inadequate. As a result, this research aimed to discover how project-based learning, semi-immersive virtual reality, and mental health influence learning performance. For data collection, 800 questionnaires were distributed to high schools, and 290 valid questionnaires were considered for the data analysis using Smart PLS-SEM. According to the study's findings, project-based learning is critical for improving learning performance. This study's findings are significant because they show that mental health and semi-immersive virtual reality significantly mediate the relationship between project-based learning and learning performance. The study's findings are critical for educational institutions interested in improving student performance through project-based learning opportunities. Furthermore, because it employs an innovative technology acceptance model, this study has significant practical implications for project-based learning and student learning performance.

## Introduction

The ICT era has been changing the traditional learning dynamics by providing alternatives to the students for better learning and performance. Teachers' dominance with a centralized curriculum, high student obedience, memorizing culture, and exam-oriented motivations are a few highly criticized education cultures causing mental health problems in youth. Such a narrowly followed educational system is also why students drop out while outperforming on international academic grounds (1). According to some researchers, focusing on students' learning performance on international grounds or in comparison with international students is still a complex discussion. Because of a lack of modern and technological learning resources provided to students in early education, they may be unable to meet specific standards (2). Due to Confucian education culture, student performance outcomes in early education are low. This learning tradition forces students to strive to learn and achieve high grades. Teachers and parents are only concerned with hard work and high grades, and the students' sole goal is to pass the examination. Because they are only exposed to the taught curriculums, their learning performance based on the new and innovative learning methods is low in this context. Moreover, such learning performance impacts their future educational goals, career, primary subject selection in colleges, and career goals (3). Compared to American students, the learning system used in China and the mindset growth of students in modern times are more focused on accepting new technology systems or conceptualized intelligence teaching. However, they are known to put in more effort to achieve goals so that they may perform well. As a result, it is essential to acknowledge that Chinese students are raising their performance to compete with students from the United States and Canada in international universities (4). However, in Chinese educational institutions, adequate resources are not provided for students to improve their learning performance with the assistance of effective and reliable teachers (5).

In this regard, the responsibility of the institutes is to provide reliable resources and develop the student's cognitive and mental abilities to improve their learning performance effectively. Indeed, the students willing to improve their performance to the advanced level are more motivated and are getting instruction from the teachers by utilizing different tools of information communication technology (ICT) to improve their learning to the advanced level (6). Also, students face a different kind of problem when competing with the students of Western countries because they are not provided with adequate training to effectively utilize ICT and its different components, such as virtual reality (7). Therefore, the student's performance is not justifiable according to the level of the students of the Western and Scandinavian countries. Different critics think that the Chinese government is not providing appropriate resources, and the stakeholders of the educational institutes are also creating a problem for the students by limiting their performance (8). Project-based learning may be an appropriate initiative for improving students' learning performance. Students can outperform if they strongly desire to achieve their goals and use active learning techniques. This system is standard in higher international studies, but resource implementation and ability development can produce unexpected results. Project-based learning is a type of learning in which a project with a specific outcome is assigned to different people for learning performance (9). Project-based learning can help students improve their learning performance. The most widely used learning mechanism is project-based learning based on modern technological knowledge and experimental learning. Students who graduate without project-based learning lack cognitive skills, making them less productive and intellectual (10). Students learning to outperform and achieve grades have a more fearful mind, and societal pressures from teachers and parents to achieve a high score are having a traumatic effect on them. The fearful mind fails to achieve its goals, and as a result, it develops psychotic issues, commonly leading to mental health problems. Students competing for academic positions on an international scale may have more mental health issues, leading to poor learning performance (11). However, some people believe that mental stress is necessary for high achievers. Another essential factor influencing Chinese students' learning performance is the complexity of mental health and stressors. Furthermore, mental health refers to a state of mind conducive to performing productive tasks (12). People with better mental health learn more effectively than those with poor mental health. Due to semi-immersive virtual reality, people can communicate more effectively at school and work. It is a learning technology that was created with the help of the information technology sector. It is critical to recognize that student performance is directly related to the context and situation of learning. If students are given the right resources and a better learning environment, their performance will improve and develop more critical thinking skills (13). However, project-based learning is the method to divide the students into groups and provide them with a project to address effectively by communicating with each other (14). Indeed, this critical way of project solving and learning is reasonable for the students who are willing to learn and develop their skills to the advanced level. However, on the contrary, the students with better mental health perform well compared to those with average mental health (15).

The students using modern learning tools perform far better than those working on the traditional way of learning (16). In reality, the student's performance is based on the combined effort of the teacher and the student. The study aimed to understand the critical role of project-based learning, semi-immersive virtual reality, and mental health in learning performance. Interestingly, there was a clear gap in the literature that was not addressed by any earlier studies (17). In this regard, this study was designed to address this theoretical gap to improve students' performance in different educational institutes in China. This study aimed to discuss the critical and exciting role of mental health in improving learners' performance in preschool education with the idea of technology acceptance. The earlier studies were reviewed, but no earlier study was conducted to effectively discuss this significant literature gap. Therefore, this study provides the significant relationship between different variables in a significant way that would be useful for future studies to understand the relationship and develop hypotheses.

It is essential to know that the purpose of this study was to address theoretical and practical gaps to improve the performance of students studying in various educational institutes in China. In this regard, it is critical to recognize that this study provides significant theoretical implications that contribute significantly to the literature because no previous study was conducted to develop the relationship between the various variables considered in this study. Significantly, the study's practical implications must be considered to improve the learning performance of students using the project-based learning method. Moreover, the significant implications of this study would help future studies and the educational institutes of China to work in a reliable and best way for the students to improve their performance. Furthermore, this study has important practical implications for the relationship between project-based learning and students' learning performance with the aid of innovative technology acceptance. This way, the student's performance would be raised to the level required to compete with students from Western countries (18).

## Literature review

### Project-based learning and learning performance

Students are not provided adequate resources and technologies to make them learn in the educational sector due to the limited resources available and the lack of attention from the institute management and the concerned government department of any country (4). The student's performance directly depends on their interaction with the teacher and the other students in the class. Moreover, active students are improving their learning status; they are more concerned with learning innovatively and designing different strategies to get better learning opportunities in an understanding way (10). Besides, traditional methods in the education sector are no longer workable, adversely affecting students' critical thinking ability because traditional methods cannot provide complete information about essential study concepts, thus lacking credibility (19). The management of schools is responsible because they are held liable for not arranging adequate resources for the better learning of the students. Indeed, it is a fact that the students who are advanced in learning and want to furnish their skills for better productivity are more concerned with innovative ways of developing the concepts at an advanced level (20). On the contrary, students in advanced and developed countries can improve their skills and learning capabilities through technology because technology plays a critical role in people's advancement and ease of life. More appropriate opportunities and learning apparatus would be provided to the students, and as a result, the student's critical thinking and performance would be developed to an advanced level because they are always concerned with learning new technologies innovatively (21). In America, most educational institutes have developed technological innovation and integration with the learning material to provide the appropriate and reasonable method for students' better learning and understanding (22). School management's responsibility is to ensure that the students are divided into groups and that they are working with the interaction of their fellow students (15).

The students who are more interested in learning and are provided with the right opportunities work harder to improve their performance and achieve prosperity in their learning. Unfortunately, the educational institutes of backward and third-world countries have failed to integrate the new and innovative technology to ease the learning material for the students (18). The educational institutes have minimal resources, including their countries' resources. In this way, the responsibility of the management is to provide the right resources, including the right technology, to ensure that the students are familiar with the technology and that they have the appropriate and reasonable information to utilize that technology for the innovation of new methods for adequate learning. Learning is an art, and the students are directly connected to learning with their cognitive behavior and interest (18). The student is responsible for interacting with the other fellows in the class and effectively discussing the course material concept to define the best strategies for working in the class (10). In this way, it is the responsibility of the management and the teachers to divide the students into different groups and provide the study material for their critical thinking and critically understanding of the topics. Therefore, the students with better critical learning ability are more interested in interacting with the other students because they always want to learn new things with innovative and effective learning methods in groups. It is reasonable to understand that, in group learning, the students are provided with the opportunity to discuss their ideas and do a critical commentary to achieve a better and more reasonable justification (6, 20). Therefore, the study proposed the following hypothesis:

***H***_**1**_*. There is a significant relationship between project-based learning and learning performance*.

### Semi-immersive virtual reality, project-based learning, and learning performance

The traditional learning methods are outdated, and the teacher and the student collectively demand a new learning method. It is critical to understand that the students concerned with their learning are more interested in learning with the help of innovative technology in modern times (23). Technology has changed traditional learning methods and has helped improve the learning process for teachers and students. In the traditional time, the outdated and old learning method was appropriate for the students because they were provided with their learning material and courses effectively with the help of the methods of that time (19). However, technological innovation is easy to access, while the traditional ways of learning are changed and replaced with advanced and modern ways of learning. It allowed students to learn and integrate the technology in groups (24). It is critical to understand that, for the students who are provided with the appropriate technology are more active in the performance than the other students who are not using the technology (25). It is essential to understand that, with the help of innovative technologies and easy accessibility in the educational sector, the traditional learning pattern is outdated because the students are more concerned about innovative material and gadgets for learning. In the same way, with the help of digital technology, the students are provided with different kinds of alternative strategies to learn in the class environment (26). Indeed, during a pandemic, students could not get into the school and work in groups because they were not provided with the opportunity to interact socially with the class students (19). Interestingly, in the time of the pandemic, the traditional and outdated way of learning was replaced with the model learning in which the ICT was utilized to the advanced level to help people learn best (27). The services of online learning material are provided to the students to ensure they are provided with the correct information for the right situation to make the appropriate critical decisions for developing the organization to the advanced level (28).

Similarly, it is the responsibility of the management of educational institutes to ensure that the students are provided with ICT gadgets to improve their learning and performance. In America and Canada, students are utilizing virtual reality gadgets. With the help of the internet, they are improving their learning standard to an advanced level because it is essential to understand that, with the help of virtual reality, group interaction can be enhanced to ensure that the communication between the students is reliable (12, 23). Similarly, in the educational institutes of Japan, the students are provided with appropriate digital and virtual reality gadgets that are important to consider productively to ensure that the students are getting the right opportunity for learning in a developed way (27). At the same time, it is the responsibility of the students to learn about virtual reality and utilize it protectively to ensure that all the related information of group work and project-based learning is solved with the help of communication and effective measurements. In some educational institutes in China, the teachers are not more concerned about virtual reality and the gadgets related to information communication and technology (ICT) because the teachers are not well versed in motivating and providing the related knowledge to the student about virtual reality and its utilization in learning (23). However, in 50% of educational institutes in China, the internet is considered the most helpful factor for providing information to students in their advanced level training (24, 29). Similarly, in the students' group work, the teacher's responsibility is to provide the reliable and best alternative way of learning to the students according to the recommendation and requirements of modern times. The students provided with the right opportunities for learning with the help of ICT are more innovative and perform better. Therefore, the management is responsible for ensuring technologically equipped teachers for better learning of students (30).

***H***_**2**_*. Semi-immersive virtual reality moderates the relationship between project-based learning and learning performance*.

### Mental health, project-based learning, and learning performance

Mental health is considered one of the influential factors in the student's performance in any educational sector. It is critical to understand that some students are mentally strong and are better at cognitive understanding to develop concepts related to the course material (31, 32). However, on the contrary, some students are not doing well in cognitive understanding of the concepts of the study because they are not innovative, and their performance is not appropriate in group work. Indeed, the critical role of mental health is appropriate for students to learn innovatively (33). It is the responsibility of educational institutes to provide a reliable and understandable working environment to the students to ensure their performance in the educational sector (34). Moreover, the students are required to do exercises, think positively, and critically relate to the course material because it helps them work innovatively and develop strategies for learning (32). In China and America, the students are enrolled in mental exercise classes in which they are provided with practical mental training and therapy to ensure that they develop the concepts and are mentally stable to perform well in groups in project-based learning (35). On the one hand, the student's responsibility is to ensure that they are learning persuasively and cannot provide irrelevant study material to their other fellows (32). At the same time, it is also essential to understand that the students must be provided the right opportunity and working environment in project-based learning by the teacher to improve students' critical cognitive abilities (36, 37). In comparison, if the students are not tolerating the opponent's lives when working on the project-based learning material in the group, then it would not be appropriate for them to develop their concepts effectively related to their course material and the effectiveness of the information sharing in the group.

Furthermore, in Denmark's educational institutes, students are directed to learn the course material in groups to reach a significant conclusion through discussion and appropriate learning (38). Although students' critical thinking abilities develop to an advanced level, they are also effectively working when they are instructed to learn and draw a conclusion based on any project provided for group discussion (39). Furthermore, it is a reasonable opportunity for students to improve their learning performance and provide a long-term way to develop their critical thinking and performance.

Moreover, students' performance in project-based learning has highlighted that a good working environment enhances their cognitive abilities to perform better in their respective groups (31, 40). In the educational institutes of South Korea, the weekdays are considered necessary for mental ability improvement and cognitive learning of the students because they provide the right opportunity for the students to develop a better understanding effectively related to the course material (32, 41). Similarly, Japanese teachers are more concerned with providing reliable and appropriate information to the students according to their mental level (42, 43). It is essential to understand that, if the students are not protected and provided with the right opportunities according to their mental level in group learning, their performance would be declined, and they will not be capable of dealing with the critical issues in learning. The students with poor mental health and who are not attractive to the teachers will not participate actively in the class to learn the course material but present for their face showing in the only. Also, the government must develop a policy for the students to improve their mental ability and cognitive concept learning environment with the help of effective teachers to support the innovation methods (44, 45). Therefore, based on the above discussion, the following hypothesis has been developed:

***H***_**3**_**.** *Mental health moderates the relationship between project-based learning and learning performance*.

### Theory and framework development

**Figure 1:**
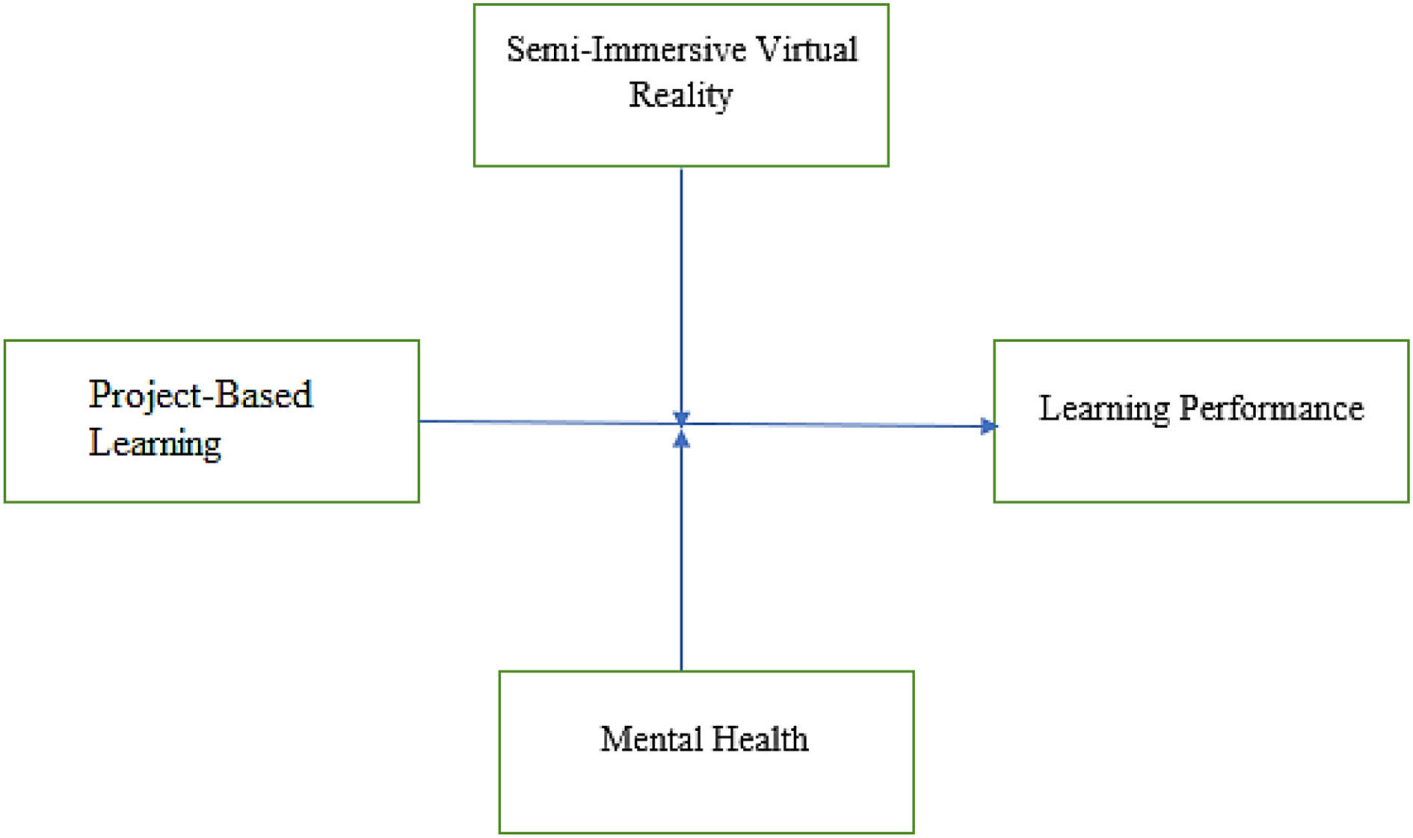
Theoretical framework.

The study's theoretical framework is grounded on the theory of learning performance. It is essential to understand that the theory of learning performance deals with the different factors influencing a learner to learn effectively and reliably (46, 47). In this regard, this theory highlights the five critical factors that contribute to and are influential in the learning performance of the students (3, 48). This theory highlights that identity is a critical factor in improving an individual student's learning performance. Second, this study highlights that learning skills are essential to improving learning performance. Third, the level of knowledge is vital to consider for the improvement of the learning performance of any student. Fourth, the critical role of the context of an individual and the knowledge for better learning improved the performance of the students. Lastly, this study demonstrates the critical role of the personal factors of every individual in the performance and learning of the individuals (49). However, during the literature review, it was identified that several other factors also contribute to the student's learning performance. In this way, the study's theoretical framework considers the critical role of mental health as a moderator and the vital role of semi-immersive virtual reality as a moderator in the relationship between project-based learning and student performance. Significantly, the results of this study would justify the moderators' vital role in improving students' learning performance in China. Furthermore, the study is based on a theoretical acceptance model, which emphasizes that individual behavior is crucial in triggering technology acceptance. In this regard, the variables considered in the study's theoretical framework are analyzed under the shadow of the technology acceptance model. The theoretical framework of the study is visual in Figure 1.

## Methodology and measures

The questionnaire for this study was prepared on a five-point Likert scale because the survey-based method is appropriate for collecting primary data from the target respondents while utilizing the least amount of cost and time. In this regard, the questionnaire was created using the Likert scale because it is simple to understand for a larger audience. In previous studies, the survey-based questionnaire was recommended as the best method for collecting data from the targeted respondents within the specified time frame (47). Notably, the scale items for each variable in this study were carefully adapted from other studies to effectively collect data from the target respondents. The scale items for mental health were taken from the study of Akbaba et al. (50). These scale items were taken to understand the relationship and role of mental help as a moderator in the relationship between project-based learning and learning performance. In previous studies, the relationship between different factors of learning performance was tested with the scale items and the quantitative data. Second, the scale items for semi-immersive virtual reality were taken from the study of Makransky et al. (23). These scale items were taken to determine the relationship and test the moderator's hypothesis in the study's theoretical framework. The role of the scale items was to collect the data from the target respondents of the study in an effective way that must be easy to understand. Third, the scale items for project-based learning were taken from the study of Akbaba et al. (50). These scale items were also carefully considered to ensure that, in the previous studies, these scale items were to measure the relationship of project-based learning with Lalit performance. Lastly, the scale items for learning performance were taken from the study of Bhattacharyya (13). These scale items were taken to understand how the learner's performance affects any individual student in the proper context of learning. Moreover, after integrating all the scale items of the study into a single questionnaire, the questionnaire was reviewed two times by different expert researchers to check the face validity of the questionnaire. Notably, after getting a positive response from the expert researchers, the questionnaire was provided to the respondents to collect the data for this study.

### Data collection

The data collection process from the target respondents is presented in detail in the study section. The study's respondents were students from China's public schools. It was difficult to obtain responses from all students; therefore, the random sampling technique effectively collected data from the target respondents. Furthermore, 800 questionnaires were distributed to the different respondents of the schools to collect the data to check the relationship between different hypotheses of the study. Notably, the individuals were asked for their permission before being given the questionnaire to complete. A detailed introduction to the study was also provided to the respondents to clarify the relationship between the study's various variables and to better understand it. Likewise, the respondents were assured that all of their data would be protected and any third party would only use it for research purposes. As a result, with an expected response rate of 40%, only 300 questionnaires were collected from the target respondents. Furthermore, after careful consideration, 290 responses were used for the study's final result.

## Findings

In this study, for the normality test, skewness and kurtosis values were determined (see Table 1). In this regard, when the value is not below +1.0, the distribution is right-skewed, and when the value is not greater than −1.0, the distribution is left-skewed (50, 51). On the contrary, for kurtosis, when the value is not less than +1.0, the distribution is leptokurtic, and when the value is not greater than −1.0, the distribution is platykurtic (52, 53). Therefore, the kurtosis and skewness of this study are normal.

**Table 1:** Skewness and kurtosis.

**Constructs**	**No**.	**Missing**	**Mean**	**Standard deviation**	**Excess kurtosis**	**Skewness**
MH1	1	0	3.895	0.968	0.503	−0.847
MH2	2	0	3.69	1.08	−0.245	−0.627
MH3	3	0	3.488	1.155	−0.56	−0.489
MH4	4	0	3.919	0.989	0.756	−0.981
LP1	5	0	4.093	0.822	1.331	−1.157
LP2	6	0	3.813	1.004	0.191	−0.768
LP3	7	0	3.771	1.134	−0.2	−0.75
LP4	8	0	3.596	1.106	−0.512	−0.467
PBL1	9	0	3.608	1.178	−0.535	−0.533
PBL2	10	0	3.729	1.092	−0.379	−0.562
PBL3	11	0	3.69	1.077	0.06	−0.651
PBL4	12	0	3.97	0.915	1.394	−1.079
SIVR1	13	0	3.747	1.01	−0.445	−0.445
SIVR2	14	0	3.958	0.968	0.05	−0.695
SIVR3	15	0	3.831	0.932	0.26	−0.666

MH, mental health; SIVR, semi-immersive virtual reality; PBL, project-based learning; and LP, learning performance.

### Convergent validity

The reliability and validity were tested to check the relationship between different variables used in the study with the help of factor loading, Cronbach's alpha, composite reliability (CR), and average variance extracted (AVE). The values are available in Table 2. In this regard, PLS algorithm calculations were considered to check the reliability and validity of the scale item used in this study to collect the data from the target respondents.

**Table 2:** Constructs, factor loadings, CR, and AVE.

**Variables**		**Constructs**	**Loadings**	**Alpha**	**CR**	**AVE**
Learning performance	LP1	I enjoy learning about new topics.	0.708	0.726	0.827	0.546
	LP2	I like to read diverse topics.	0.772			
	LP3	I find pleasure in learning.	0.787			
	LP4	I feel very good when I know I have outperformed other students.	0.682			
Mental health	MH1	I am calm and balanced in studies.	0.756	0.707	0.817	0.528
	MH2	I manage well to learn effectively.	0.683			
	MH3	All in all, I am satisfied with my studies.	0.799			
	MH4	In general, I am confident.	0.660			
Project based learning	PBL1	I expect myself to support the group's goals.	0.887	0.856	0.906	0.710
	PBL2	I expect to complete my assignments for the group on time.	0.914			
	PBL3	I expect that I will contribute to the group's success.	0.904			
	PBL4	I expect to learn more on my own than from other group members.	0.634			
Semi-immersive virtual reality	SIVR1	I was completely captivated by the virtual world.	0.834	0.836	0.90	0.751
	SIVR2	I felt like it was real in virtual reality.	0.870			
	SIVR3	I had a sense of acting in virtual environment.	0.895			

MH, mental health; SIVR, semi-immersive virtual reality; PBL, project-based learning; and LP, learning performance.

According to the results, the factor loading value for each scale item was more significant than 0.60, which is recommended by Henseler and Fassott (54), for modern studies. Similarly, the composite reliability values were more significant than 0.70, which is also recommended by Hair et al. (55), for modern studies. Values of AVE of each construct were identified and found above the threshold values of 0.50 (56). As a result, there were apparent reliability and validity between the scale items used for this study to collect the data from the target respondents with the help of a survey-based questionnaire. Furthermore, the scale items with low factor loadings were deleted in the study's data analysis.

### Discriminant validity

In this section of the study, the results of discriminant validity are presented in Table 3. It is critical to understand that the discriminant validity between the scale items of the study is to check the relationship between different scale items for different variables. The discriminant validity (HTMT) values for each variable were not >0.90 (57). Similarly, all the discriminant validity values were according to modern studies' requirements. This study has an apparent discriminant validity between the scale items utilized to collect the data from target respondents and measure it to test the relationship between different study variables.

**Table 3:** Discriminant validity (heterotrait–monotrait—HTMT).

	**LP**	**MH**	**PBL**	**SIVR**
**LP**				
**MH**	0.814			
**PBL**	0.836	0.864		
**SIVR**	0.765	0.54	0.425	

MH, mental health; SIVR, semi-immersive virtual reality; PBL, project-based learning; and LP, learning performance.

### The partial least square structural equation modeling (SEM)

**Figure 2:**
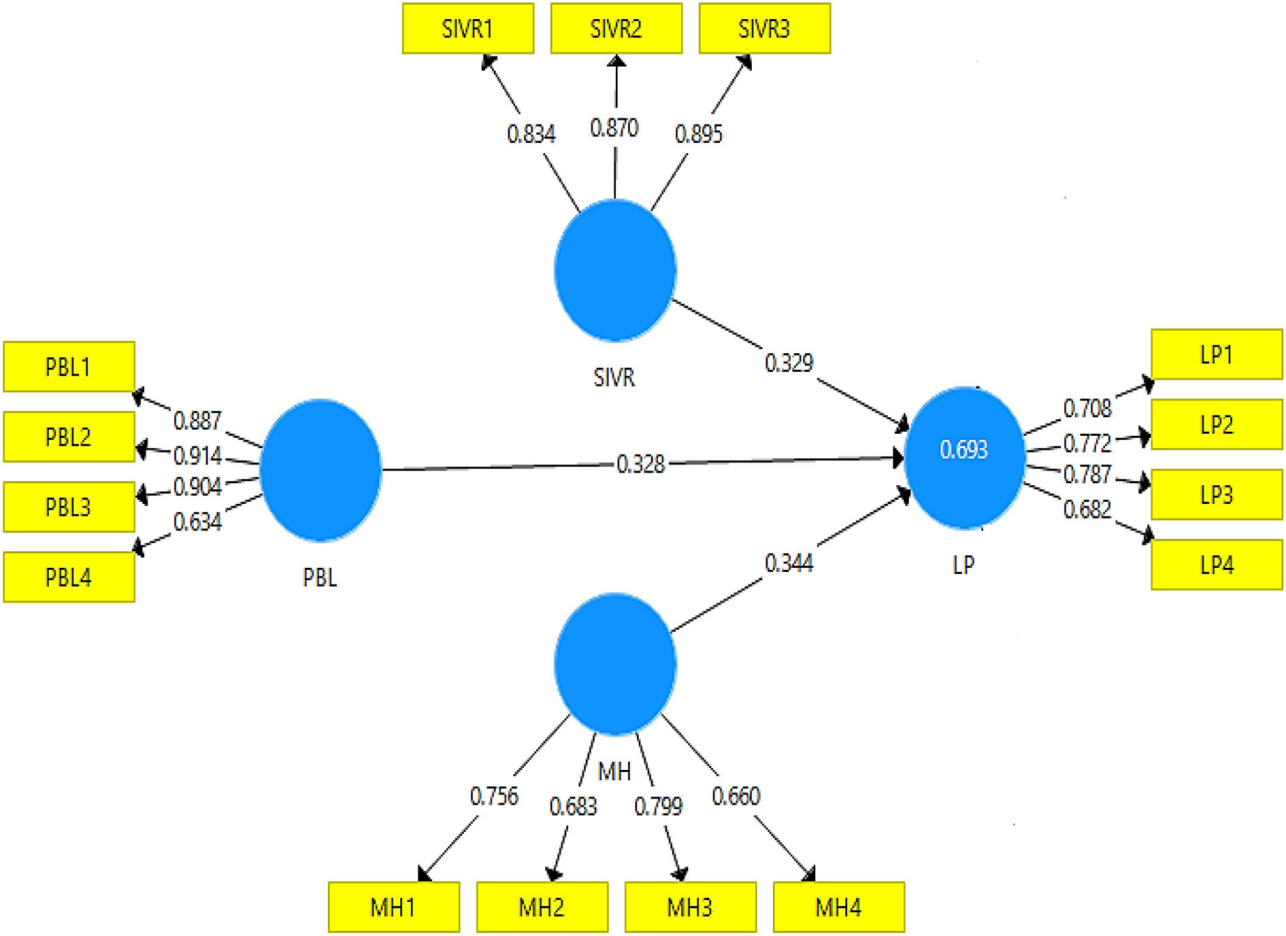
Measurement model. MH, mental health; SIVR, semi-immersive virtual reality; PBL, project-based learning; and LP, learning performance.

**Figure 3:**
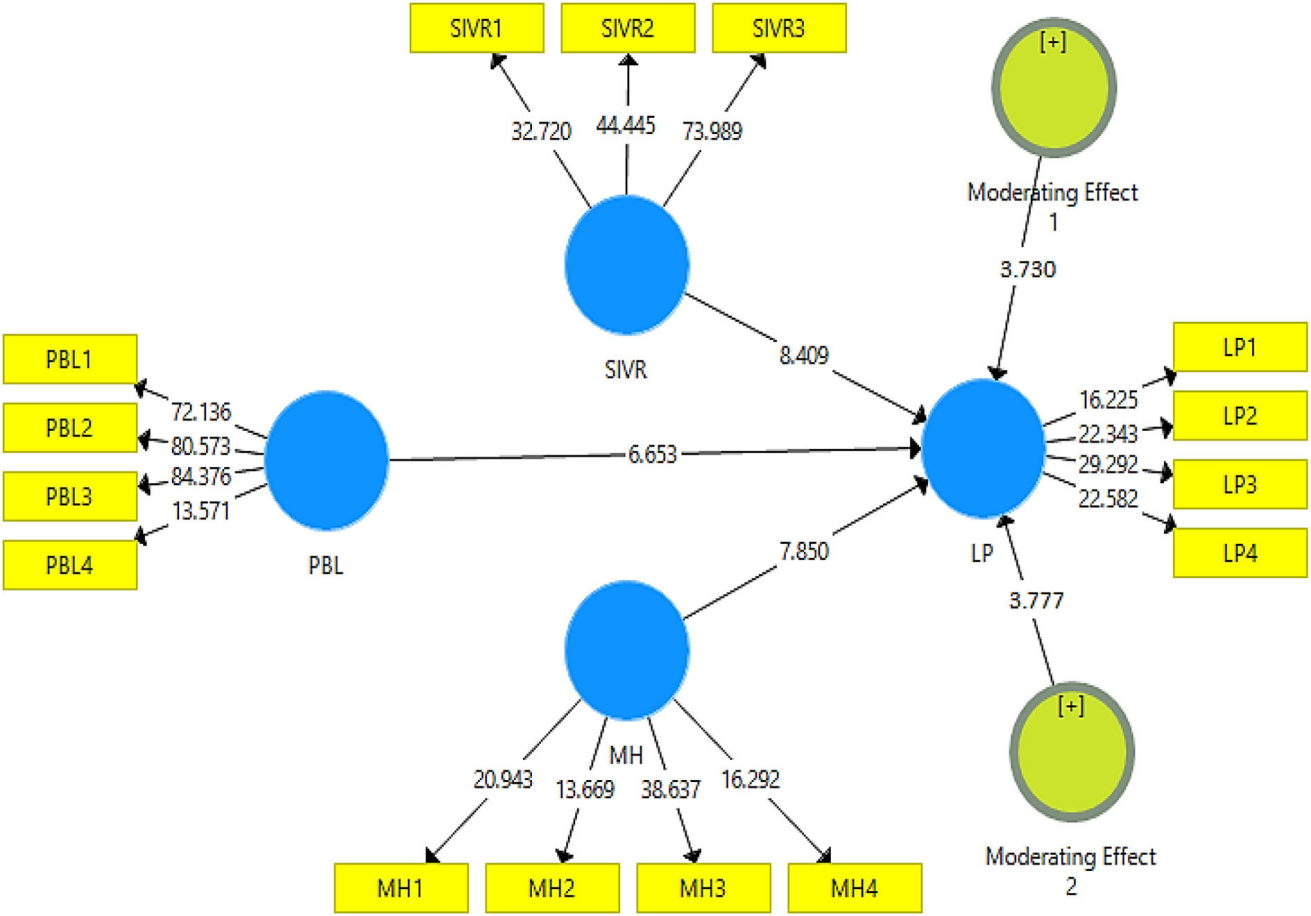
Structural model. MH, mental health; SIVR, semi-immersive virtual reality; PBL, project-based learning; and LP, learning performance.

The hypotheses of this study were tested with the help of partial least square structural equation modeling results, which are visible in Figures 2, 3. In this regard, according to values in Table 4, hypothesis 1 was tested, and the results revealed a significant relationship between project-based learning and learning performance (β = 0.328, *t* = 6.653, and *p* = 0.000). Furthermore, hypothesis 2 was tested, and the results revealed the significant moderating role of semi-immersive virtual reality in the relationship between project-based learning and learning performance (β = 0.097, *t* = 3.730, and *p* = 0.000). Lastly, hypothesis 3 was tested, and the results revealed the significant moderating role of mental health in the relationship between project-based learning and learning performance (β = 0.068, *t* = 3.777, and *p* = 0.000). The results of the hypotheses tests are available in Table 5.

**Table 4:** Hypothesis testing.

**No**	**Relationship**	**Hypotheses**	**Beta**	**STDEV**	***T*-values**	***P*-values**	**Remarks**
**1**	Direct	PBL -> LP	0.328	0.049	6.653	0.000	Significant

PBL, project-based learning; and LP, learning performance.

**Table 5:** Hypothesis moderation testing.

**No**	**Relationship**	**Hypotheses**	**Beta**	**STDEV**	***T*-values**	***P*-values**	**Remarks**
**2**	Moderation	Moderating Effect 1 -> LP	0.097	0.026	3.730	0.000	Significant
**3**	Moderation	Moderating Effect 2 -> LP	0.068	0.018	3.777	0.000	Significant

LP, learning performance.

**Figure 4:**
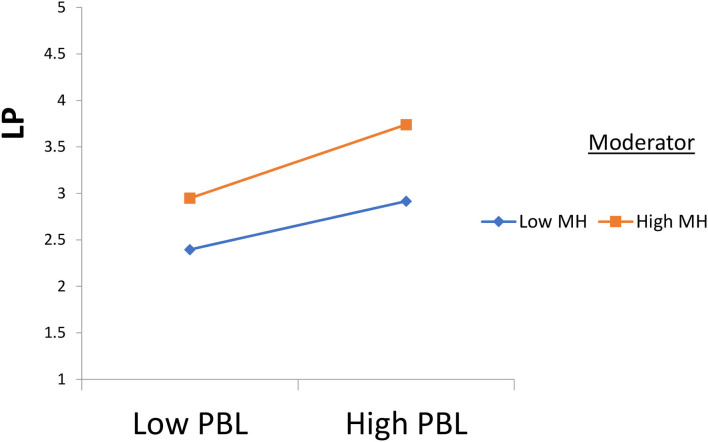
Moderation 1. MH, mental health; SIVR, semi-immersive virtual reality; PBL, project-based learning; and LP, learning performance.

**Figure 5:**
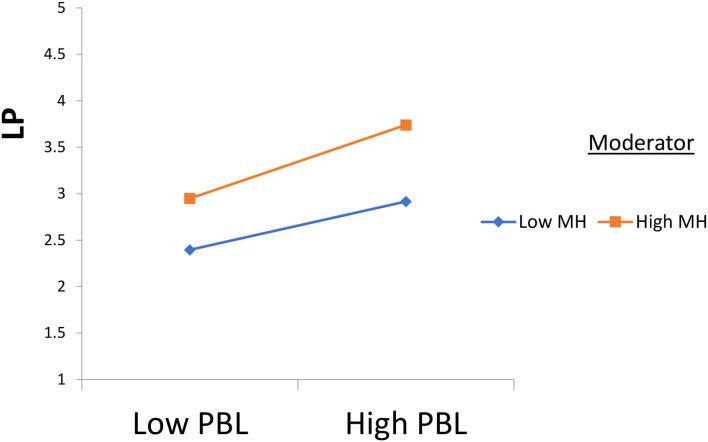
Moderation 2. MH, Mmental Hhealth; SIVR, Ssemi-Iimmersive Vvirtual Rreality; PBL, Pproject-Bbased Llearning; and LP = Llearning Pperformance.

Furthermore, according to Figure 4, semi-immersive virtual reality strengthens and positively moderates the relationship between project-based learning and learning performance. Also, according to Figure 5, mental health strengthens and positively moderates the relationship between project-based learning and learning performance.

### Predictive relevance

In this study, to check predictive relevance in variables of the study, the PLS blindfolding calculator was used to identify values. Hair et al. recommended that the value of *Q*^2^ should be >0 to make the model predictive (58). The findings of *Q*^2^ highlight a predictive relevance among the variables of research. Table 6 of the research explains the predictive relevance of the study because *Q*^2^ is >0.

**Table 6:** Predictive relevance.

**Constructs**	**SSO**	**SSE**	***Q*^2^ (=1-SSE/SSO)**
LP	1,328	878.107	0.339
MH	1,328	1,328	
PBL	1,328	1,328	
SIVR	996	996	

MH, mental health; SIVR, semi-immersive virtual reality; PBL, project-based learning; and LP, learning performance.

## Discussion

According to hypothesis 1, there is a significant relationship between project-based learning and learning performance because it improves students' skills and allows them to learn and practice more in training to meet their goals. Indeed, the students working in groups under the project-based learning strategy provide good performance to develop the conclusion and justify it according to the context of the situation (31). The performing ability measures can be easily predicted in project-based learning by dividing the students into homogeneous groups and providing deadline-based tasks demonstrating the ability of learning performance and student achievement capacity. To improve the performance of the student's learning, teachers must divide the students into groups, give them a deadline for achieving the target objective, and reach a conclusion by discussing the course material and the related problems with effective communication and understanding. (56). In this regard, the students who are provided with the opportunity of such kind are credible to improve their learning performance effectively to enhance their experience (57). Moreover, the responsibility of the educational institutes is to utilize the new and innovative way of learning for the better learning performance of the students (58). Therefore, the more reasonable the working environment provided by the teachers, the more productivity the students would develop to improve their learning performance. Significantly, in the educational institutes of America, the students are directed to learn the course material in a group to reach a significant conclusion with the help of discussion and appropriate learning (59, 60). In this way, not only does students' critical thinking ability develop to an advanced level but also does the students ability to effectively work improve when they are instructed to learn and draw a conclusion based on any project provided to discuss in the group (39). Also, it is a reasonable opportunity for the students to improve their learning performance and provide them with a sustainable way of developing their critical thinking ability and performance (61–63).

According to hypothesis 2, semi-immersive virtual reality plays a significant moderating role between project-based learning and learning performance because students who learn with technology innovation integrated systems are academically capable and have appropriate technical skills compared to those who do not with technology-integrated systems. Indeed, educational institutions are developing a strategy to advance students' performance through virtual reality and the two distinct functions provided by ICT (64–66). Students in developing countries are not given adequate opportunities to develop their critical thinking skills through ICT and other virtual reality tools (23). Indeed, it is critical to understand that virtual reality helps students to learn innovatively because visual observation helps them remember the concepts and the course material in the long term (67). Importantly, for the students that are not provided with that kind of innovative technology to learn in a better way and improve their performance, the performance students are limited, and it is not according to the level of the students who are provided with the opportunity of learning with the help of information communication technology in an effective way (57, 67, 68). It is reasonable to understand that, with the help of virtual reality, the traditional learning dynamics have been changed because the students are provided with a different alternative to learn innovatively and develop effective regulation for their better learning. On the contrary, in advanced and developed countries like America and Canada, students use virtual reality as a tool for learning, and it helps improve their performance to the appropriate level (24, 69, 70). Therefore, the student's learning performance is far better than those of the backward countries, which are not provided with appropriate resources by the stakeholders and the government policies to improve their learning standards to the advanced level.

The results of hypothesis 3 highlight a significant moderating role of mental health in the relationship between project-based learning and learning performance. It is critical to understand that, the students who are provided with the opportunity to develop their mental health effects during the class session and different exercises in the educational sector, their performance of the students is improved when they are learning in a group based on the project-based learning method (33, 40). The project-based learning method is essential for the students because it allows them to develop different strategies and reach a conclusion with the help of fellow students in a group (71). However, if a student's mental health is appropriate, the student would develop critical thinking ability and dominate the group in his views to improve the learning standard to the appropriate level and reach a conclusion (42, 56). In Japan, the students are trained to improve their mental health and are provided with reasonable exercises to develop their cognitive level according to advanced studies to ensure that they are provided the appropriate level of certification for their better learning (72). Indeed, cognitive factors and mental health work a lot in the study because, with the help of better mental health, it is easy for the students to read and draw a conclusion in the project-based learning opportunity (64, 73). Significantly, in third-world countries, the trend of mental health importance is increasing, and the educational institutes are considering it to the advanced level for the better development of the cognitive ability of the students (74, 75).

Similarly, the responsibility of the teachers is to design the curriculum in an attractive way to provide the best learning method to the students to improve their standards. Moreover, mental health defines the understanding ability of any students related to the course material (29, 31, 33). To improve mental health effectively, the students have the critical role of getting involved in different cognitive exercises provided by the experts (76, 77). In this way, the student's mental understanding would be increased, and they would perform well in their learning methods (78, 79).

## Conclusion

The study framework is based on the Chinese school and college education system, where students work hard to achieve the highest possible score to compete with international students. In this regard, there is a critical need to provide technology resources and teaching methods to improve students' technical knowledge and ability to obtain the appropriate content of modernized, innovative, and technology-based knowledge. In this study, project-based learning, mental health, and semi-immersive virtual reality were investigated in conjunction with the technology acceptance model to assess the learning performance of students in schools and colleges. The hypotheses were developed to provide helpful information. Project-based learning positively impacts learning performance because students get to know and learn different skills through different projects and develop the ability to perform practically through those developed skills. Mental health and semi-immersive virtual reality played a significant moderating role in the relationship developed between project-based learning and learning performance. When it comes to mental health, it is a crucial factor to consider because enhancing a student's healthy mind is required for project-based skills. In addition, semi-immersive virtual reality allows students to learn practically in a controlled environment. It enhances the essence of project-based learning among students and, as a result, improves their learning performance.

Teachers and parents in China are working to improve students' mental health and learning performance, but learning performance can be improved by motivating students to perform well in group-based activities for project-based learning. Surprisingly, it is the stakeholders' responsibility to integrate ICT and physical exercises to improve students' cognitive and mental abilities to improve their performance when learning in groups using the project-based learning method. Furthermore, this study highlighted the significance of project-based learning in improving learner performance because it allows students to discuss the project in groups and reach an effective conclusion to develop different strategies for better understanding and compatibility. Students' performance would improve and be developed in an effective way to use ICT in favor of learning to get better responses and adopt an easy and understandable way of learning to achieve better results.

## Implications

### Theoretical implications

This study adds new variables to the literature to improve learners' performance in China, providing a robust theoretical framework. The relationship between project-based learning and learning performance was not discussed in previous studies, despite the moderating role of mental health and semi-immersive virtual reality. In education, students enrolled in schools and colleges can embrace technological innovation because of their ability to exert effort to achieve high marks. As a result of the study's findings, stakeholders must consider the critical role of project-based learning in improving the performance of school students because adequate and dependable policies must be conducted and implemented efficiently to ensure students' prosperity and prosperity-improved learning behavior.

Furthermore, this study demonstrates that, by effectively implementing virtual reality, educational institutes in China will improve learner performance and provide them with the most reasonable way to obtain the appropriate level of learning. Better mental and cognitive abilities must also be considered to improve students' project-based learning for effective learning. The students should be allowed to improve their mental ability through various means to improve their standard of performance in learning and a better understanding of the course material when they are in a group and working on the project-based method of learning. Significantly, the study's scope is critical for teaching institutes to improve student performance by providing the right opportunities at the right time to get the correct output from the students.

### Practical implications

This study's practical implications would benefit education stakeholders and teaching staff at schools, colleges, and universities. First, we will address the sensitive issue of student mental health. As discussed in this study, it moderates the relationship between project-based learning and learning performance. Teachers must ensure that their students enrolled in project-based learning are mentally fit and gain the necessary expertise from the content taught. If students' mental health is poor, they may be unable to learn and perform well despite all the available resources. As a result, it is an essential measure for assessing students' learning performance. In practice, this study shows that, if teachers and stakeholders develop and implement appropriate policies for students' learning, the appropriate role of reasonable mental cognitive ability must be considered, and students must be provided with various types of exercises and study material to help them improve their mental health and critical thinking ability.

Students' cognitive abilities will improve with good mental health, aiding in their project-based learning and skill development. Another critical factor highlighted in this study that is required for improving learning performance is semi-immersive virtual reality. Integrating technology for innovation into the education system is insufficient if there is no practical exposure. Teachers can create a virtual reality environment where students can virtually experience and practice with various tools and gadgets to improve their comprehension and practical ability. China is a developed nation with an advanced technological system. Incorporating these technology-based systems into the educational system would improve students' mental intelligence while expanding their professional opportunities. Students learning performance in schools can be improved to a reasonable level with effective management by using the project-based innovative learning method, which also improves students' critical thinking ability. Significantly, teaching institutes must be provided with appropriate resources to improve learner performance by preparing them to learn using modern and advanced virtual reality tools. Not only would the learners' performance improve but also would the learners be given the necessary opportunities, in line with top countries where students are given these abilities and tools to learn effectively and perform well.

## Limitations and future directions

This study aimed to determine the role of semi-immersive virtual reality and mental health in Chinese students' learning performance. However, various other factors are also helping students improve their learning performance. In this regard, future research may concentrate on the role of technological acceptance for teachers in improving student performance. Similarly, future research should concentrate on the critical role of teachers' mental abilities in improving student performance. Finally, future research should focus on mobile technology's critical moderating role in improving students' learning performance using the project-based learning method. It would significantly contribute to the literature and to improve the student's learning performance for project practitioners. This study's limitation is its small sample size and 40% response rate. However, future studies must collect data from a large sample size to justify the findings.

## Data availability statement

The original contributions presented in the study are included in the article/supplementary material, further inquiries can be directed to the corresponding author/s.

## Ethics statement

The studies involving human participants were reviewed and approved by Shandong Women University, China. The patients/participants provided their written informed consent to participate in this study. The study was conducted in accordance with the Declaration of Helsinki.

## Author contributions

YK and JD conceptualized the study and collected the data. JZ wrote the draft. All authors agreed to the submitted version of the manuscript.

## Conflict of interest

The authors declare that the research was conducted in the absence of any commercial or financial relationships that could be construed as a potential conflict of interest.

## Publisher's note

All claims expressed in this article are solely those of the authors and do not necessarily represent those of their affiliated organizations, or those of the publisher, the editors and the reviewers. Any product that may be evaluated in this article, or claim that may be made by its manufacturer, is not guaranteed or endorsed by the publisher.
